# Adolescents with Type 1 Diabetes – The Impact of Gender, Age, and Health-Related Functioning on Eating Disorder Psychopathology

**DOI:** 10.1371/journal.pone.0141386

**Published:** 2015-11-03

**Authors:** Line Wisting, Lasse Bang, Torild Skrivarhaug, Knut Dahl-Jørgensen, Øyvind Rø

**Affiliations:** 1 Regional Department for Eating Disorders, Division of Mental Health and Addiction, Oslo University Hospital, Oslo, Norway; 2 The Norwegian Childhood Diabetes Registry, Department of Pediatric Medicine, Oslo University Hospital, Oslo, Norway; 3 Department of Pediatric Medicine, Oslo University Hospital, Oslo, Norway; 4 Faculty of Medicine, University of Oslo, Oslo, Norway; 5 Oslo Diabetes Research Centre, Oslo, Oslo, Norway; 6 Division of Mental Health and Addiction, Institute of Clinical Medicine, University of Oslo, Oslo, Norway; Sickle Cell Unit, JAMAICA

## Abstract

**Objective:**

To investigate correlates of eating disorder psychopathology in adolescent males and females with type 1 diabetes.

**Method:**

A total of 105 adolescents with type 1 diabetes (42% males), aged 12–20 years, were recruited from the Norwegian Childhood Diabetes Registry in this population-based study. All participants were interviewed with the Child Eating Disorder Examination. Additionally, the Brief Illness Perception Questionnaire, the Adolescent Coping Orientation for Problem Experiences and the Beliefs about Medicines Questionnaire were administered to assess health-related functioning. Clinical data were obtained from the Norwegian Childhood Diabetes Registry.

**Results:**

Significant gender differences were demonstrated in the pattern of correlates of eating disorder pathology. Among females, eating disorder psychopathology was significantly associated with body mass index adjusted for age and gender, age, insulin restriction, coping, illness perceptions, and perceptions of insulin concern. In a regression model, age, illness perceptions, and insulin restriction remained significantly associated with eating disorder psychopathology, explaining 48% of the variance. None of the variables were associated with eating disorder psychopathology among males.

**Discussion:**

Greater clinical awareness of illness perceptions, attitudes toward insulin, and insulin restriction may potentially decrease the risk of developing eating disorders among female adolescents with type 1 diabetes, and the subsequent increased morbidity and mortality associated with comorbid type 1 diabetes and eating disorders.

## Introduction

T1D appears to be a risk factor for the development of eating disorder psychopathology; with prevalence rates of eating disorders in T1D approximately twice those of healthy controls [1,2]. Symptoms of eating disorder psychopathology include dieting, binge eating, purging, weight- and shape concerns, and disturbed body image [3]. Possible contributing factors for the increased risk of developing disturbed eating in T1D include increased monitoring of food intake as part of standard T1D care and weight gain subsequent to insulin treatment [4]. One study found that adolescent females were seven kg heavier than their non-diabetic peers [5]. This can lead to increased weight- and shape concerns among young females with T1D. Furthermore, insulin restriction (reducing or omitting insulin) is a purging behavior uniquely accessible to patients with T1D, and an efficient weight loss strategy. Insulin restriction is reported in up to 32–36% of females with T1D [6–8].

T1D alone is associated with increased rates of morbidity and mortality compared to non-diabetic populations [9–11], and combined with the presence of disturbed eating, the prognosis is even worse. A study of comorbid T1D and anorexia nervosa showed that the crude mortality rate at a 10-year follow-up was 2.5% for T1D and 6.5% for anorexia nervosa, but mortality rose to 34.8% when T1D and anorexia nervosa co-occurred [12]. Also, prior research has found that self-reported insulin restriction at baseline leads to a three-fold increased risk of mortality at an 11-year follow-up [6].

Given the poor prognosis associated with comorbid T1D and disturbed eating, greater knowledge is warranted regarding the associated correlates of disturbed eating in individuals with T1D. Age and body mass index (BMI) have previously been found to be associated with eating disorder psychopathology in patients with T1D [8]. Very little is known about risk factors for the development of disturbed eating in T1D. One longitudinal study by Olmsted et al. [13] found that poorer self-esteem and higher levels of depression and body and weight concerns were associated with a higher risk of developing disturbed eating among young females with T1D. Health- related functioning, such as illness perceptions (i.e., individual perceptions or beliefs about their illness) and coping strategies (i.e., behaviors adopted to handle negative or stressful events) have been found to be relevant for outcome in various mental and somatic illnesses, including eating disorders [14–16] and T1D [17,18]. However, little is known about how these variables potentially affect disturbed eating in individuals with T1D. Furthermore, although insulin restriction is a common feature of disturbed eating in T1D, attitudes toward insulin have not previously been investigated in relation to eating disorder psychopathology in individuals with T1D. Knowledge about correlates and possible contributors to disturbed eating in T1D is important to inform prevention and treatment, as there is currently no consensus on the ideal treatment approach for this high-risk patient group.

### Aims of study

This study aimed to investigate associations between eating disorder psychopathology and illness perceptions, coping strategies, insulin beliefs, insulin restriction, body mass index, and age in young males and females with T1D. We also aimed to assess the magnitude of the explained variance in eating disorder psychopathology, and to investigate whether the pattern of associations differed by gender.

## Materials and Methods

### Procedure

The Norwegian Childhood Diabetes Registry (NCDR) is a nationwide, population-based registry, which includes all newly diagnosed children with diabetes since 1989. All pediatric departments in Norway perform and report the results of annual standardized examinations to NCDR. This cross-sectional study is part of a larger study of the NCDR, which included 850 participants (12–20 years). Between 2011 and 2012, all 850 individuals were invited to participate in an in-depth assessment of eating disorder psychopathology and health-related functioning. Of the 850 individuals who received information and consent forms, a subset of 105 individuals (12%) agreed to participate by returning a signed consent form via postal mail. The remaining 745 individuals did not respond. The assessment was conducted at Oslo University Hospital or another location of the participants’ choice (usually their home or school).

### Ethical aspects

The data protection department at Oslo University Hospital and the Regional Committees for Medical and Health Research Ethics approved the study, including consent procedure. Written informed consent was obtained from all participants and their parents if the participant was below the age of 16 years (parental consent is not required for adolescents above the age of 16). LW and LB anonymized participant data.

### Measures

#### The Child Eating Disorder Examination (ChEDE)

The ChEDE [19] is a semi-structured diagnostic interview that is considered the gold standard for assessing eating disorder psychopathology among children and adolescents. The ChEDE has been translated and validated in Norwegian and has demonstrated good psychometric properties [20]. The ChEDE consists of four subscales (restraint, eating concern, weight concern and shape concern), in addition to a global score. Due to mixed support for the original 4-factor structure [21] of the ChEDE, only the global score is used in this study as a dimensional measure of eating disorder psychopathology. The answers range from 0–6, and higher scores indicate a higher degree of eating psychopathology. In line with Olmsted et al [13], a diabetes-adapted version of the ChEDE was adopted to ensure that pathological scoring was due to weight and shape concerns, and not only for controlling the diabetes. As such, any endorsement of items related to eating behavior or food (e.g., food rules, dietary restraint, etc) was further queried to determine whether such behavior was attributable to insulin-treated diabetes or motivated by concerns about their weight and shape. Only attitudes or behaviors motivated by shape/weight concerns were rated. Additionally, following the administration of the items on bulimic episodes and overeating, the patient was asked to estimate the percentage of such episodes that were associated with a hypoglycemic/low blood sugar reaction. Finally, a separate item for insulin restriction was added, which queried whether the patients had ever reduced or omitted their insulin dose. If the patients responded affirmatively, they were asked why insulin was restricted. Interviews were conducted by two Master’s-level graduate students in psychology (LW and LB) who had participated in training seminars for the administration the ChEDE interview by its developer (Rachel Bryant-Waugh). Inter-rater reliability was assessed for subscales and global score totals and found to be good (composite intraclass correlations coefficient of .97).

#### The Brief Illness Perception Questionnaire (BIPQ)

The BIPQ [22] is a brief version of the Illness Perceptions Questionnaire (IPQ) [23] and Illness Perceptions Questionnaire–Revised (IPQ-R) [24], and is a valid and reliable measure of illness perceptions. It has been used in the context of a variety of illnesses, including T1D. It consists of nine items, and each item assesses one dimension of illness perceptions: consequences, timeline, personal control, treatment control, identity, coherence, emotional representation, concern and causation. Answers are scored on a Likert scale ranging from 0–10, and higher scores indicate more threatening/negative views of their diabetes.

#### The Beliefs about Medicines Questionnaire (BMQ)

The BMQ [25] is a measure of beliefs about medicines in general, and beliefs about one specific medicine. In this study, beliefs toward insulin were assessed. The BMQ consists of four subscales: specific (insulin in the current study) necessity, specific (insulin) concern, general necessity and general overuse. Answers range on a five-point Likert scale, from 1 = strongly disagree to 5 = strongly agree. A Norwegian version has been translated and validated, demonstrating satisfactory psychometric properties [26]. The specific (insulin) necessity- and concern subscales were used in this study to measure participants’ concerns regarding taking insulin and to what extent they perceive insulin to be necessary.

#### Adolescent Coping Orientation for Problem Experiences (A-COPE)

The ACOPE [27] is a measure of coping strategies and is translated and validated for use among Norwegian adolescents [28]. In addition to a total score indicating the degree of positive coping, the A-COPE consists of 34 items divided in five subscales: being social, seeking diversions, ventilating negative feelings, developing self reliance, and solving family problems. Answers range from 1–5 (never, seldom, sometimes, often, usually). Higher scores indicate a higher degree of positive coping on all items.

#### Clinical data

Clinical data were obtained from NCDR. *HbA1c* was determined for all participants by high-performance liquid chromatography (HPLC) (Tosoh G7; Tosoh Europe N.V., Belgium). All samples were analyzed in the same central laboratory at Aker, Oslo University Hospital, and standardized according to the Diabetes Control and Complications Trial standards. The reference range was 4.0–6.0%; the analytical coefficient of variation was < 1%.

*zBMI* is age- and gender-adjusted Body Mass Index (BMI). BMI was calculated based on weight and height (kg/m²) and standardized to a z-score according to age and gender using the Centers for Disease Control and Prevention Growth Charts 2000, as the participants were primarily below 18 years [29].

### Statistical analyses

Associations between eating disorder psychopathology, illness perceptions, coping strategies, insulin beliefs, insulin restriction, age, and zBMI were assessed by means of Spearman’s correlations (*p* < .05). In line with Cohen [30], correlations of .10 to .29 were interpreted as small, .30 to .49 as medium and .50 to 1.0 as large. Following the bivariate correlations, standard multiple regression (all independent variables entered simultaneously) analyses was conducted using a the backward elimination strategy and an alpha level of *p* < .05 to investigate possible risk factors for eating disorder psychopathology. The analyses were split by gender. Group differences were investigated using t-tests. Pearson chi-squares were used for categorical variables. Intraclass correlation analysis was used to assess inter-rater reliability. Effect sizes were calculated by means of Cohen’s *d*, and the guidelines used for interpreting this value were: .20 = small effect, .50 = moderate effect and .80 = large effect [30]. Statistical analyses were conducted using PASW version 21 (SPSS IBM, NY, USA).

## Results

Table 1 illustrates sample characteristics. Participants resided in rural and urban settings across all geographical regions of Norway. A total of 65.3% of the participants used insulin pumps and 33.7% used pen. There were 44 (41.9%) males and 61 (58.1%) females. Age range was 12–20 years. Male and female participants did not differ significantly on age (yrs), HbA1c, zBMI, age of onset, duration of diabetes illness (yrs), or mode of treatment (pump versus pen). Participants were compared to the background T1D population in the NCDR, which has a completeness of 95% [31]. These groups did not differ in age, zBMI (age- and gender adjusted body mass index), T1D duration, number of consultations with the diabetes team, number of consultations with dieticians, or mode of treatment. Participants were slightly older at the onset of T1D than the background NCDR population (9.6 versus 8.8 years, *p*< .05), had somewhat lower HbA1c (8.6% (70 mmol/mol) versus 8.9% (74 mmol/mol), p < .05), and had fewer episodes of diabetes ketoacidoses (.02 versus .05, *p* < .05). However, the effect sizes were small (.2, -.2 and -.2, respectively).

**Table 1:** Sample characteristics.

	All	Males	Females	p-value
n	105	44	61	
Age (years)	15.7 (1.8)	15.9 (1.8)	15.6 (1.8)	ns
HbA1c (%)	8.6 (1.3)	8.4 (1.3)	8.7 (1.3)	ns
zBMI	0.4 (0.8)	0.3 (0.8)	0.4 (0.9)	ns
Diabetes duration (years)	5.7 (3.7)	5.7 (3.6)	5.7 (3.7)	ns
Age at onset of diabetes (years)	9.6 (3.5)	9.8 (3.6)	9.5 (3.5)	ns
Eating pathology (ChEDE global score)	0.7 (1.0)	0.2 (0.4)	1.0 (1.8)	.001
Insulin pump	65.3%	59.5%	69.5%	ns
Insulin pen	33.7%	38.1%	30.5%	ns

Note: Data represent the mean (SD). ChEDE = Child Eating Disorder Examination

The correlation matrix is presented in Table 2. Among females, the ChEDE global score was significantly associated with zBMI (*p* < .05), age (*p* < .01), insulin restriction due to weight and shape concern (*p* < .01), the ACOPE total score (*p* < .05), the BIPQ overall score (*p* < .01), and the BMQ subscale insulin concern (*p* < .01). None of the variables were significantly associated with the ChEDE global score among males.

**Table 2:** Correlations between eating disorder psychopathology and illness perceptions (BIPQ), coping strategies (ACOPE), insulin beliefs (BMQ), insulin restriction due to weight- and shape concerns, age and zBMI.

				Females (n = 61)					
		ChEDE global	BIPQ overall	ACOPE total	BMQ insulin necessity	BMQ insulin concern	Insulin restriction	Age	zBMI
	ChEDE	—-	**.416** ***	**-.313** *	**.124**	**.452** ***	**.387** **	**.452** ***	**.311** *
	BIPQ	-.057	—-	**-.348** *	**-.058**	**.354** **	**.346** **	**.125**	**.129**
	ACOPE	.182	.093	—-	**-.153**	**-.197**	**-.302** *	**-.203**	**-.073**
Males (n = 44)	BMQ insulin necessity	.151	-.176	.074	—-	**-.203**	**-.263** *	**.154**	**.091**
	BMQ insulin concern	.151	.115	.049	-.351*	—-	**.360** **	**.299** *	**.174**
	Insulin restriction	-	-	-	-	-	—-	**.206**	**.138**
	Age	.272	-.250	.134	.314*	-.256	-	—-	**.210**
	zBMI	.212	-.028	.015	.144	-.069	-	.110	—-

Note: Significance level

* = < .05

** = < .01

*** = < .001.

No cases of insulin restriction due to weight- or shape concern among males. BIPQ = Brief Illness Perception Questionnaire (overall score); BMQ = Beliefs about Medicines Questionnaire; ChEDE = Child Eating Disorder Examination (global score); ACOPE = Adolescent Coping Orientation for Problem Experiences (total score).

Associations between the variables were further investigated in a regression analysis (Table 3). Among females, the ACOPE total score, the BIPQ overall score, BMQ insulin concern, insulin restriction, age, and zBMI were entered into the regression equation. This model explained 53% of the variance in the ChEDE global score (*p* < .0001). In line with the backward elimination strategy, the least significant variables were removed one by one until only significant variables remained. Age (β = .32, *p* < .01), the BIPQ overall score (β = .26, *p* < .05), and insulin restriction due to weight concern (β = .40, *p* < .001) remained significantly associated with the ChEDE global score in this regression model, explaining 48% of the variance.

**Table 3:** Regression model for females, with eating pathology as the dependent variable.

	B	Std. Error B	β	t	Sig
Age	.209	.066	.316	3.168	.003
BIPQ overall score	.027	.011	.255	2.418	.019
Insulin restriction due to weight and shape concern	.040	.011	.401	3.785	.000

Note: R^2^: .48 (p < .0001)

BIPQ = Brief Illness Perception Questionnaire

Due to no significant associations with the ChEDE global scores among males, no regression analysis was conducted in this group.

## Discussion

This study found significant gender differences in the pattern of correlates associated with eating disorder pathology. Among females, insulin restriction, age, and illness perceptions were significantly associated with eating pathology, explaining 48% of the total variance. None of the variables were significantly associated with eating disorder psychopathology among males.

Insulin restriction was relatively strongly associated with eating disorder psychopathology among females. This is in line with existing literature describing insulin restriction as a core feature of disturbed eating in T1D [6], and an efficient weight loss strategy and compensatory mechanism which is uniquely available to individuals with T1D. Insulin is the primary treatment of T1D and sufficient administration of insulin is essential to achieve recommended treatment targets and ensure good health [32]. Insulin restriction is found to be associated with poor metabolic control, as well as increased risk of diabetes morbidity and mortality [6]. In our study, beliefs about insulin (i.e., insulin concern) were also significantly correlated with eating pathology. Stronger perceptions of insulin concern (i.e., concerns about taking insulin) were associated with higher levels of insulin restriction and eating pathology. This unique finding appears to make clinical sense given the frequency of insulin restriction as a method of weight loss among females with T1D [7]. To our knowledge, no prior studies have investigated beliefs about insulin in relation to insulin restriction and eating pathology in T1D. The present findings suggest that assessment and awareness of behaviors and attitudes toward insulin are central to understanding disturbed eating in T1D, and might be worth addressing clinically, for example by using the specific subscales of the Beliefs about Medicines Questionnaire as a starting point for further conversations.

The impact of age on eating pathology is in line with previous research in both T1D and non-T1D populations, as levels of eating pathology have been found to increase during adolescence [33,34]. For example, our previous study found that the prevalence of disturbed eating behavior was five times higher among females aged 17–19 years than young females aged 11–13 years [8]. Similarly, the prevalence rates among pre-teen and early teen girls with T1D generally appear to be lower than the prevalence rates reported in studies of older adolescent and young adult samples with T1D [7,35].

Illness perceptions have been found to be related to metabolic control in adolescents with T1D [18]. With regard to eating pathology, a systematic review [36] of illness perceptions in adults reported that illness perceptions were associated with various health outcomes. Specifically, greater illness-related distress was associated with a higher degree of anxiety, depression, and general psychological distress. Additionally, illness perceptions were found to be associated with readiness to change, indicating the importance of information about illness perceptions in guiding treatment approach. Collectively, illness perceptions seem to be important to a variety of health-related outcomes. To our knowledge, our study is the first to specifically investigate the association between illness perceptions on eating pathology in individuals with T1D. It is possible that the development of eating pathology serves a (dysfunctional) way of coping with the challenges of adapting to a chronic illness and the potential negative impact on daily life.

Our data revealed significant gender differences in various domains. While the variables for health-related functioning were relatively strongly correlated with eating disorder psychopathology among females, none of the variables were significantly associated with eating disorder psychopathology among males. Although our data cannot explain these gender differences further, one possible explanation for the lack of significant correlations among males is that the males in this study had significantly lower degrees of eating pathology than females. However, the gender differences in health-related functioning was also evident in our previous study [37] investigating correlates of metabolic control (HbA1c). There were no significant differences between males and females in metabolic control, indicating that gender differences in health-related functioning in this study may have other explanations than solely less eating pathology. Gender differences with regard to psychosocial factors and eating pathology are rarely explored in existing research on T1D, as the literature on eating disorders has traditionally focused on females. This is probably due to the lower prevalence rates of eating pathology among males, and limited access to treatment for male patients with eating disorders, both in patients with T1D and the normal population. More research is needed to further investigate the prevalence and correlates of eating pathology in male adolescents.

Strengths of this study include the face-to-face assessment conducted with the Child Eating Disorder Examination on a relatively large sample of adolescents with T1D. The participants were not solely recruited from one T1D clinic, but from many hospitals around Norway across a large geographic area. This study is strengthened by the collaboration with the Norwegian Childhood Diabetes Registry (NCDR), allowing for comparison between participants and non-participants. The participants were very similar to the population as a whole, facilitating generalizability. Finally, our study included male adolescents, which to date represents an understudied population in the field of eating disorders.

The cross-sectional design is a limitation of this study. Longitudinal studies are needed to investigate causal relationships and identify specific risk factors for the development of disturbed eating in T1D. Our data suggest that health-related functioning should be included in future research on risk factors for the development of disturbed eating in T1D among adolescent females. Increased knowledge about the risk factors for the development of eating pathology in T1D would inform prevention and treatment efforts for this at-risk patient group. This study is the latest of several studies that has recruited patients from the NCDR. Although the response rate of 12% represents a limitation of this study, it is strengthened by the collaboration with the NCDR that ensures clinical data is quality-controlled and complete for 95% of the total population of young patients with T1D in Norway [31]. This enables comparison of participants to non-participants on various clinical and demographical data. In general, few significant differences were found between our participants and the non-participants, except for somewhat higher age at T1D onset, slightly lower HbA1c levels and somewhat fewer episodes of diabetes ketoacidoses. In addition, a higher percentage of our participants used insulin pump compared to the T1D background population in the NCDR. This might indicate that our subgroup is slightly healthier than the rest of the population. Despite small effects, however, this is considered a limitation of the current study.

Several clinical and research implications can be drawn from this study. Insulin concern and insulin restriction was relatively strongly associated with eating pathology among females. Additionally, illness perceptions generally proved to be important in explaining the variance in the amount of eating pathology. Increased clinical attention and awareness of illness perceptions may help inform prevention or treatment efforts to decrease the risk of developing the challenging comorbidity of T1D and eating disorders. Patient perceptions and illness beliefs also deserve greater research attention using longitudinal designs. Relevant questionnaires, such as the Brief Illness Perception Questionnaire, may be used as a starting point to facilitate further conversation with patients. Efforts to reduce negative perceptions about T1D and its treatment may positively impact on disturbed eating behaviors. Based on the results of this study, differential treatment approaches for male and female adolescents who struggle with disturbed eating and T1D might be warranted, but more research is needed to investigate this further. Clinicians should be vigilant to the risk of developing disturbed eating in patients with T1D, especially among young females.
